# Central Regulation of Eating Behaviors in Humans: Evidence from Functional Neuroimaging Studies

**DOI:** 10.3390/nu15133010

**Published:** 2023-07-01

**Authors:** Younbyoung Chae, In-Seon Lee

**Affiliations:** College of Korean Medicine, Kyung Hee University, Seoul 02447, Republic of Korea; ybchae@khu.ac.kr

**Keywords:** eating behavior, neuroimaging, cognitive modulation, obesity, eating disorders

## Abstract

Neuroimaging has great potential to provide insight into the neural response to food stimuli. Remarkable advances have been made in understanding the neural activity underlying food perception, not only in normal eating but also in obesity, eating disorders, and disorders of gut–brain interaction in recent decades. In addition to the abnormal brain function in patients with eating disorders compared to healthy controls, new therapies, such as neurofeedback and neurostimulation techniques, have been developed that target the malfunctioning brain regions in patients with eating disorders based on the results of neuroimaging studies. In this review, we present an overview of early and more recent research on the central processing and regulation of eating behavior in healthy and patient populations. In order to better understand the relationship between the gut and the brain as well as the neural mechanisms underlying abnormal ingestive behaviors, we also provide suggestions for future directions to enhance our current methods used in food-related neuroimaging studies.

## 1. Introduction

Ingestive behavior, which encompasses all feeding and drinking behaviors, is a complicated process that involves gut–brain signals, homeostatic and hedonic mechanisms, cognitive processes, and social and psychological factors that influence what, when, and how much we eat [1,2]. The excessive consumption of high-calorie foods and abnormal eating behaviors, such as unnecessary dietary restraint and binge eating, have become major public health concerns worldwide [3]. Therefore, a number of functional neuroimaging studies have been conducted to identify the cognitive perception of and neural responses to food-related information in healthy and patient populations.

Eating disorders (EDs), obesity, and disorders of gut–brain interaction (DGBI), previously known as functional gastrointestinal disorders, are life-threatening conditions with high morbidity and mortality. However, the basic mechanisms underlying these conditions have rarely been investigated. Patients with irregular eating behaviors experience severe psychiatric disturbances, psychological distortions, and somatic conditions that negatively affect their daily lives [4]. Understanding where harmful eating behaviors originate and why the response to food is distorted in affected patients would improve the current approach to the management of these disorders. Neuroimaging studies could contribute to characterizing the essential role of the brain in the modulation of eating behavior [5,6].

For example, functional magnetic resonance imaging (fMRI), which measures neural activity indirectly using blood-oxygen-level-dependent (BOLD) signals, is completely non-invasive and has high spatial (a few millimeters) and acceptable temporal (a few seconds) resolution. Deep brain structure and brain stem imaging are also possible. However, the fMRI environment can be challenging because participants must remain motionless in the scanner for 20–60 minutes. It also has some limitations, most of which are related to head motion and metal objects. Positron emission tomography (PET) is a minimally invasive imaging technique that uses radiotracers injected into participants to measure the metabolic distribution of the radiotracers in the brain. Depending on the radiotracers used, it can measure various metabolic activities in the brain, e.g., blood flow using ^15^O-H_2_O, glucose metabolism using ^18^F-fluorodeoxyglucose, and dopamine receptor activity using ^11^C-raclopride and ^11^C-methylspiperone. Magnetoencephalography (MEG) is completely non-invasive and provides participants with a more comfortable environment (silent and sitting positions are available). It measures neuronal magnetic activity with high temporal resolution (milliseconds) but low spatial resolution (a few millimeters to a centimeter). Functional near-infrared spectroscopy (fNIRS) is a relatively new technique that examines cerebral activities using near-infrared light. It has the advantages of safety, low cost, portability, and moderate temporal and spatial resolution. However, it can only measure the activity of superficial layers of brain tissue indirectly using oxygenated and deoxygenated hemoglobin (see [6,7] for more details).

Although structural neuroimaging studies have contributed to our knowledge of human ingestive behavior (e.g., reduced orbitofrontal cortex [OFC] volume in obesity [8], and decreased gray matter volume in anorexia nervosa [AN] [9]), we have restricted the scope of this review to functional neuroimaging studies and brain functions related to eating behavior in healthy individuals and patients with an ED, obesity, and DGBI.

## 2. Eating Behavior and Brain Functions

### 2.1. Energy Homeostasis, Reward, and Food Craving

**Figure 1:**
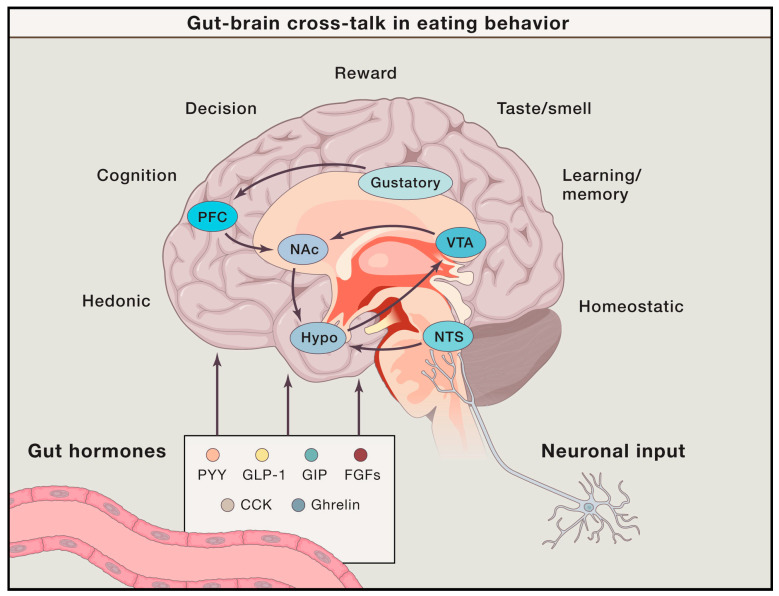
The interaction of cognitive factors, brain functions, and gut hormones influences human eating behavior. Homeostatic brain regions (e.g., hypothalamus, nucleus tactus solitaries) are responsible for processing food intake and glucose levels, whereas hedonic regions (e.g., nucleus accumbens, ventral tegmental area, and prefrontal cortex) mediate reward-related food-eating behavior. These brain regions also contain hormone receptors and respond to hormonal changes released by the gut, influencing eating behavior. CCK: cholecystokinin; FGFRs: fibroblast growth factor receptors; GIP: glucose-dependent insulinotropic polypeptide; GLP-1: glucagon-like peptide-1; PFC: prefrontal cortex; PYY: peptide YY; NAc: nucleus accumbens; VTA: ventral tegmental area; Hypo: hypothalamus; NTS: nucleus tractus solitaries. Reprinted from Publication Cell, 168/5, Christoffer Clemmensen,Timo D.Müller, Stephen C. Woods, Hans-Rudolf Berthoud, Randy J. Seeley, Matthias H. Tschöp, Gut–Brain Cross-Talk in Metabolic Control, Pages No. 758–774, Copyright (2017), with permission from Elsevier [1].

The central nervous system controls food intake through complementary cooperation between the homeostatic control system and the food reward system, which involves the synthesis of gastrointestinal hormones, such as peptide YY, ghrelin, leptin, and insulin [1,10] (Figure 1). For instance, the homeostatic system, centered in the hypothalamus, including the ventromedial and lateral hypothalamus [11] and arcuate nucleus [12], is responsible for adjusting energy intake in response to the fuel level of the body. Increased extracellular glucose levels excite glucose-sensing neurons in the medial hypothalamus and inhibit the feeding center neurons in the lateral hypothalamus [13,14]. Glucose-sensitive neurons are also found in the hindbrain [15,16] and reward systems (amygdala, hippocampus, and nucleus accumbens [NAc]) [17]. In addition, ghrelin receptors in the hypothalamus, brainstem, and reward pathways mediate food intake and glucose metabolism [18,19].

The consumption of palatable foods, even when additional energy is not required, indicates that food is rewarding. The hedonic reward system in the brain (dopaminergic system in the ventral tegmental area, striatum, NAc, amygdala, and prefrontal cortex [PFC]) mediates reward-related food-eating behavior. For example, dopaminergic activity in the reward system has been consistently observed during the anticipation of food-related rewards [20,21] and in response to palatable and caloric foods [22,23,24,25], and may override homeostatic control and interact with the control of food intake [10,26]. However, this does not necessarily indicate that food intake behavior is solely dependent on central reward processing. Leptin, insulin, and ghrelin act directly on dopaminergic neurons and affect the reward system in the brain [27,28,29]. In addition, there is little but convincing evidence regarding the critical role of the brain–gut axis in the reward processing of food. For example, Salamone et al. showed that food intake in rats was not inhibited by the ablation of dopamine neurons that project to the NAc, but it reduced their desire to work hard for food rewards [30]. Scholtz et al. also demonstrated that patients with obesity showed significantly reduced responses to high-calorie food images in the brain reward system after gastric bypass surgery compared to patients with obesity who did not undergo surgery [31]. In a recent review, Araujo et al. suggested a model in which conscious hedonic qualities seem less important in food reinforcement. Instead, gut–brain reward pathways [32] work independently of cognitive processes and bypass neural networks that give rise to food perception and reinforcing behaviors [33]. In other words, habit formation and reinforcement can occur naturally, even in the absence of conscious taste appreciation, suggesting that subliminal reward signals in the gut may play a significant role in eating behavior [34,35].

In a healthy population, appetite was found to be suppressed after meals because postprandial satiety signals change the secretion of gut hormones [36] and gastric distention signals [37]. Nutrients entering the gut influence brain activities through the brain–gut axis (communication between the enteric nervous system and central nervous system [38]), which results in the perception of fullness and satiation. Food cravings make food appetizing, causing the desire for and consumption of food. Pelchat et al. conducted the first fMRI study to examine how the brain responds to food cravings induced by an imagery task and found craving-related activation in the insula, caudate, and hippocampus of healthy participants [39]. Recently, Koban et al. identified an fMRI-based neurobiological craving signature, a neural pattern that can be a potential biomarker for craving, which can predict the self-reported intensity of drug and food cravings in the ventromedial PFC, striatum, thalamus, and cingulate cortices [40]. Among the EDs, binge eating disorder (BED) and obesity have been the main focus of studies on food cravings, specifically in terms of failure to control food cravings. Rolls and McCabe showed that people who crave chocolate present a significantly higher activation in the OFC, ventral striatum, and pregenual cingulate cortex with the sight and flavor of chocolate than non-cravers [41]. The role of food craving and its influence on brain function in patients with functional dyspepsia (FD) have also been studied. In addition to increased anxiety and depression, patients with FD showed significantly higher food craving state scores compared to healthy controls. Moreover, this increased craving in patients with FD inhibited the resting-state brain activity in the middle frontal gyrus, with depression as a mediator [42]. Although patients with FD showed a greater craving for food than healthy controls, they showed a decreased total processing time for food images, suggesting that increased craving does not necessarily increase attentional responses [43].

In summary, eating behavior in healthy populations and patients with an ED is influenced by the central processing of reward, the hedonic value of foods, the gut-originated reward pathway, and the craving for food. These elements may interact with psychological factors, such as anxiety and depression, and the gut environment may also have an impact on how food properties are processed in the brain.

### 2.2. Appetite Control and Cognitive Inhibition of Eating Behavior

It is also noteworthy that eating behavior is not passively determined by energy demand and hormonal changes. We modify our behavior to achieve a certain goal, and cognitive modulation makes our behavior more flexible. Thus, impairments in cognitive functions, such as deficits in inhibitory functions, lead to abnormal eating behaviors, such as binge eating and food avoidance; however, the role of cognitive processes has only recently been recognized. PFC regions are related to executive function, inhibitory control, and the integration of reward signals [44,45]. For example, satiation by eating a meal significantly reduced brain activity in reward-related regions, while increasing brain activity in the dorsolateral PFC and enhancing functional connectivity between the dorsolateral and ventromedial PFC, suggesting an effect of satiety signals on top-down cognitive control in the brain [46]. Hare et al. showed that when dieters exercised self-control during food-related decision-making tasks (choosing which food to eat, either healthy or nutritionally inferior), activity in the ventromedial PFC correlated with goal values (overall food values that combine health and taste considerations) regardless of the amount of self-control, and the dorsolateral PFC was more activated in successful self-controlled choice trials (choosing food based on both health and taste considerations) than in non-self-controlled choice trials (choosing food solely based on taste value). They suggested that the dorsolateral PFC affects self-control by modulating value integration in the ventromedial PFC [44]. Previous studies have shown that patients with acute AN show increased dorsolateral PFC activity during the presentation of visual food cues [47], which may persist even in populations that have recovered from AN [48]. In addition, obesity is associated with a lack of impulse control, which is closely related to inhibitory control and reward processing. For example, obese individuals show a greater delay in discounting, preferring to accept small immediate rewards (food and money) [49,50]. An fNIRS study discovered decreased PFC activity in obese patients and patients with both obesity and BED, compared to healthy controls, while viewing food images and performing a Go/NoGo task. The result implies abnormal inhibitory control in these patients, which could be related to impulsivity [51].

Abnormal addictive behaviors toward food in patients with an ED are related to reward circuitry, which is also responsible for substance and non-substance addiction [52]. By assessing and modulating abnormal brain functions involved in appetite control and reward, we might help patients with obesity control their eating behaviors. For example, patients with bulimia nervosa (BN) and BED show significantly higher food craving traits than healthy controls, and increased craving for food is positively correlated with brain activity in the medial OFC, the region responsible for processing reward value and feelings of pleasantness. In cocaine-addicted participants, the medial OFC showed increased brain metabolism in response to drug cues [53,54] and food rewards [55]. Several studies have shown that structural and functional abnormalities of the OFC are associated with obesity [56]. Rolls et al. analyzed brain functional connectivity data during rest in a large population (*n* = 37,286) and showed that functional connectivity between the ventromedial PFC/OFC and anterior cingulate cortex (ACC) was positively correlated with the preference for sweet foods and body mass index (BMI). In addition, OFC functional connectivity, with a number of other frontal cortex areas, was higher relative to other brain areas in the high BMI group than in the mid-range BMI group [57]. These results suggest that the functions of the intrinsic neural circuitry may influence affective responses to food, even when food-related cues are not presented, and may cause an increase in BMI. It is important to determine whether this individual variation is congenital or due to repeated exposure, dietary education, or lifestyle. Furthermore, as both sensory and cognitive factors can activate the reward system in the brain, it is crucial to understand how and whether these brain abnormalities can be recovered in patients with an ED.

In summary, the reward value of food, food cravings, appetite control, and cognitive inhibition are closely related to eating behavior. EDs and obesity involve neural circuitries of reward processing and cognitive control of food and non-food reward stimuli (e.g., money). Neuroimaging studies of ingestive behavior allow us to reveal the underlying mechanisms of EDs and highlight potential steps for the treatment of people with an ED. For example, exploring the neurological pathways of appetite control is critical for the treatment of overeating. Neurostimulation therapy may be a potential way to help patients with an ED because impaired executive function and reward processing are typically not resolved in recovered or remitted patients [48,58] and are largely ignored in the treatment of patients with an ED, contributing to the high chronicity and subsequent public health burden of these conditions.

### 2.3. Functional Brain Activities Elicited by Real Food Consumption

The neural mechanisms underlying the perception of solid food have been less studied. In most studies, liquid-type solutions have been used owing to the technical limitations of the neuroimaging environment [59]. For example, among the first neuroimaging studies incorporating food-related stimuli were PET and fMRI studies that used tastants to evaluate the brain processing of taste [60,61]. A recent meta-analysis found that liquid tastants and liquid-type foods (juice, wine, milkshakes, etc.) significantly activated clusters in the insula, precentral gyrus, caudate, thalamus, and middle and posterior cingulate cortices. In addition, regarding the affective, intensity, and quality domains of taste, the middle insula is a crucial area for food and taste perception [59]. Liquid-type solutions have mostly been used to investigate the neural processing of tastes (sweet, bitter, salty, etc.) and nutrients (fat, caffeine, sucrose, glucose, etc.) [62,63,64,65,66,67,68,69]. Neuroimaging studies using liquid-type tastants have demonstrated taste quality-related [64] and intensity-related neural representations in the brain [63]. Avery et al. used 7-tesla fMRI and multivariate searchlight analyses to study how the brain discriminates sweet, salty, and sour tastes. Multivariate analysis identified distinct neural patterns for each taste in sensory processing (somatosensory cortex and mid-insula), reward, and affect-related regions (striatum, orbitofrontal cortex, thalamus, and amygdala), although univariate analyses failed to identify distinct neural representations for each taste [64]. According to Canna et al., who tested five concentrations of both pleasant (sweet) and unpleasant (bitter) tastes, the mid-posterior insula processes taste intensities independent of the quality and valence of the tastants [63].

Neuroimaging studies using liquid-type foods have investigated the effects of satiety [65] and fasting using chocolate milk and chicken broth [66], texture and fat using oil [67], and reward processing using juice, milk [62], and milkshakes [68,69]. Solid and soft foods have also been used in imaging studies; in the majority of these instances, neural activity was recorded and compared before (fasting state) and after meal intake to investigate the effect of meal consumption on hormonal or neural signal changes [42,70,71,72,73]. For example, using PET, Small et al. found that dopamine release in the dorsal striatum was significantly correlated with the pleasantness rating of a favorite meal using PET [71]. Frank-Podlech et al. tested how fat content affects cerebral blood flow [73] and the functional connectivity between the brain stem, hypothalamus, and reward-related regions [72] using high- and low-fat meals. They found that high-fat yogurt reduced cerebral blood flow in the hypothalamus more than low-fat yogurt [73] and that fat content interacts with oral fat sensitivity regarding functional connectivity patterns between homeostatic and reward regions [72]. In addition, the functional connectivity between the insula and occipital cortex significantly increased after consuming high-fat yogurt, whereas it significantly decreased after consuming low-fat yogurt in patients with FD [42]. Compared with lean participants, NG et al. found that patients with obesity showed significantly greater activity in the Rolandic operculum and ventromedial PFC during regular milkshake consumption than during the consumption of identical low-fat milkshakes [70]. These results suggest that the consumption of a nutrient influences brain activity and that the processing of food-related information in the brain is affected by oral sensitivity and cognitive processing of nutritional factors.

In conclusion, solid or liquid food types have been used to study how the brain processes information about food, including taste, texture, nutrients, and the influence of cognitive factors. Although each factor’s primary processing regions have been identified, such as the dorsal striatum for food-related pleasantness and the insula for taste intensity and quality, more research is still needed to understand how these regions interact.

### 2.4. Functional Brain Activities Elicited by Food Images and Food Imagery

According to a recent paper that evaluated 23 neuroimaging meta-analysis papers, 11 of the 23 meta-analyses included real food stimuli, while more studies included either visual food images (*n* = 14) or tastants (*n* = 13) [74]. Visual food cues have been used more frequently to investigate ingestive behavior in healthy individuals and patients in neuroimaging studies, as a wider range of foods, including both liquid and solid, can be presented compared to studies using real food stimuli. The validity of incorporating imaginary tasks using visual cues to study the central mechanisms of food perception has been questioned. However, evidence supports that viewing food images involves the brain regions associated with saliency [75], taste perception [76], hunger and satiety states [77], and reward processing (e.g., OFC, ACC, and ventral striatum) [78,79]. Zheng et al. applied an activation likelihood estimation (ALE) meta-analysis method to identify brain regions that respond to images of low- and high-calorie foods in participants with a normal weight. They found that the left OFC, amygdala, insula, superior parietal lobe, and fusiform gyrus were activated when viewing high-calorie foods, whereas the left fusiform gyrus was activated when viewing low-calorie foods [80]. In addition, several studies have shown that brain activity in response to food images is altered in patients with an ED. Brooks et al. applied an ALE meta-analysis and showed that patients with obesity had reduced dorsolateral PFC and insular activity while viewing food images [81]. Using the same approach, Zhu et al. showed that patients with AN showed significantly greater activity in the caudate, frontal and cingulate gyrus and significantly lower activity in the somatosensory cortex, precuneus, inferior/superior parietal cortex while viewing food pictures, compared to healthy controls [82]. In a recent fMRI study using a multivariate pattern analysis approach, Avery et al. demonstrated that both viewing and imagining the taste of images of food activated the middle and ventral-anterior insula, and clusters in the middle insula showed reliable classification performance to classify gustatory tastes (sweet, salty, and sour) and the taste of food pictures (sweet, salty, and sour). The results suggest that food images could activate similar brain responses as taste perception of food; however, the patterns of neural activity are distinguishable between visual food information and gustatory information, as well as between different qualities of taste [83].

Kobayashi et al. conducted an fMRI study to investigate the top-down processing of gustation using gustatory perception (fruit juice) and gustatory imagery tasks (thinking about taste in the absence of actual taste stimuli). They found that gustatory imagery and perception commonly activate the insula and precentral gyrus and that the middle and superior frontal gyri participate in the generation of gustatory hallucinations by retrieving gustatory information [84].

In conclusion, visual food images have been extensively used in neuroimaging studies of food-related behavior because they offer more varied stimulation options. Additionally, using food cues and tasks requiring imagination, cognitive assessment and emotional reactions to foods can be assessed. However, more research must be conducted to look at more usual eating behaviors, if the delivery of food to participants is not hindered by technical issues.

### 2.5. Neuroimaging Studies in Eating Disorders, Obesity, and Disorders of Gut–Brain Interaction

#### 2.5.1. Anorexia Nervosa (AN)

Neuroimaging studies have found altered activation in the hypothalamus, amygdala, and insula during passive viewing of food image tasks in patients with AN compared with healthy participants [85,86]. Patients with AN reported less hunger and desire to eat food than healthy controls, and cortisol levels were correlated with both appetite and activity of the hypothalamus, amygdala, and insula [86]. An fMRI and diffusion-weighted imaging study investigated structural and functional effective connectivity in patients with AN during a sucrose-tasting task and found reduced structural connectivity between the OFC and hypothalamus, and effective connectivity was directed from the PFC to the ACC, the ACC to the ventral striatum, ventral striatum, and hypothalamus [87]. These results suggest that patients with AN have disrupted homeostatic and hedonic behavior and neural signals in response to food. Furthermore, the results suggest that the circuitry between the homeostatic and reward evaluation centers is abnormal in patients with AN compared to healthy controls.

Since a key strength of neuroimaging is that it can investigate the neural representations of psychological processes, some studies have focused on how patients with AN perceive food images and found that the right middle frontal gyrus and frontal pole were significantly activated more than in healthy participants (while imagining eating food [88] and thinking about the presented high-calorie foods, respectively [89]). In contrast, the frontal pole showed decreased activity in patients with AN compared to healthy participants when thinking about low-calorie foods [89]. These results indicate that the top-down cognitive control system in patients with AN is impaired, which might be related to the inhibition of food intake. The frontostriatal circuit has been studied in patients with AN and suggested as a target region for brain stimulation treatments [90]. An fMRI study showed that patients with AN chose a smaller portion of high-fat foods than healthy controls and that the food choice task was related to brain activity in the dorsal striatum. Functional connectivity was associated with actual caloric intake in the AN [91], and the results suggest that impaired goal-directed decision-making processes (e.g., maladaptive food choice even in a state of starvation) in the AN are associated with malfunctioning of the frontostriatal pathway.

A clinical study involving repetitive transcranial magnetic stimulation (TMS) targeting the dorsolateral PFC in patients with AN showed that treatment significantly improved AN and reward-related behaviors [92]. More recent studies also found that repetitive TMS to the dorsolateral prefrontal cortex significantly reduced self-controlled food choices (i.e., choosing healthy, less tasty foods or not choosing unhealthy, tasty foods) and increased tasty but unhealthy food choices [93].

In summary, the decreased reward value of food and food avoidance behavior of patients with AN might be related to abnormalities in the reward and cognitive control systems in the brain. Recently, a new approach targeting the brain itself has been applied to treat their food choice behaviors. However, further studies are required to directly link the behavior and brain function of patients with AN and to investigate the mechanisms underlying the recovery of symptoms.

#### 2.5.2. Bulimia Nervosa (BN)

The frontostriatal circuit plays a critical role in patients with BN-like AN. As functional connectivity between the dorsolateral PFC and dorsal striatum is related to food choice behavior in patients with AN [91], this circuit might also be related to disturbed self-control of food intake behavior, resulting in binge eating in patients with BN. For example, an fMRI study investigated the self-regulatory aspects of patients with BN using the Simon spatial incompatibility task, which ignores task-irrelevant information while focusing on task-relevant information. They found that patients with BN showed significant deactivation in the inferior, medial, and superior frontal gyri during the task, whereas healthy controls showed significant activation in the frontal cortices, putamen, and hippocampus. The results suggest that patients with BN failed to engage the frontostriatal pathway to inhibit their behavior when they were presented with conflicting priorities [94,95] (see [96] for more detailed review).

In addition to the deficit in self-regulation, brain regions involved in food processing and food-related reward learning showed different activities in patients with BN than in healthy controls. For example, patients with BN were asked to think about eating the presented food, and they showed decreased activation in the insula and superior temporal gyrus compared to healthy controls [97]. Bohon and Stice measured BOLD signals while patients with BN anticipated or received either a chocolate milkshake or tasteless control solution. They found that patients with BN showed less activity in the precentral gyrus during the reward (chocolate milkshake) anticipatory phase and less activity in the middle frontal gyrus, precentral gyrus, insula, and thalamus (regions that did not survive correction for multiple comparisons) [98]. In addition, Frank et al. demonstrated reduced BOLD signals in the insula, putamen, and OFC in response to expected or unexpected tastes in patients with BN compared with healthy controls using a classical conditioning paradigm [99]. Moreover, abnormal reward systems might be restricted to food stimuli, as patients with BN and BED showed similar brain activities to healthy controls in response to the monetary reward task [55]. A single-session clinical trial showed the potential effect of transcranial direct current stimulation (tDCS) on self-regulatory behavior and mood in patients with BN [100]; however, the effects need further clarification due to conflicting evidence [101].

In summary, dysfunction of the top-down control system and attenuated neuronal responses to food and food-related reward processing might be related to binge eating episodes and the consequent compensatory behavior in BN.

#### 2.5.3. Binge Eating Disorder (BED)

Although patients with both BN and BED experience binge episodes, neuroimaging studies have revealed overlapping and distinct neurobiological mechanisms underlying BN and BED. Patients with BED and BN showed decreased posterior cingulate cortex (PCC) activity during the anticipatory phase of food reward (expectation of high reward versus expectation of low reward) and increased activities in the PCC and OFC during the receipt phase of food reward compared to healthy controls [55]. According to pattern decoding analysis, patterns of brain activation in the insula successfully distinguished patients with BED (accuracy = 0.86) and BN (accuracy = 0.78) from healthy controls, and patterns in the ventral striatum showed the highest classification accuracy for the classification between patients with BED and BN (accuracy = 0.84) [102]. These results suggest that patients with BED show impaired reward sensitivity in the brain, similar to patients with BN; however, the neural patterns between patients with BED and BN differ.

In contrast to BN, BED is closely related to obesity, as compensatory behaviors to prevent weight gain have not been observed. Hege et al. found higher attentional impulsivity scores in obese and overweight patients with BED than in BMI-matched control participants without BED, and the higher attentional impulsiveness was related to decreased inhibitory response during a Go/NoGo task, as well as to hypoactivity in prefrontal networks, which was measured with MEG, for successful no-go withhold trials [103]. Brain activity in the PFC, measured via fNIRS, was also reduced during the inhibitory control tasks compared to healthy controls [51], but increased after impulsivity-focused cognitive behavioral therapy [104]. The findings suggest that in patients with BED, insufficient ability to activate the PFC may be restored after behavioral treatment. Burgess et al. also demonstrated the effects of tDCS on BED, food cravings, and preferred food intake [105], suggesting a potential role for neurostimulation treatment in impaired inhibitory control in patients with BED.

In summary, previous studies show that, in addition to disrupted reward sensitivity, attentional impulsiveness and failure of frontal activity are responsible for impaired inhibitory control in patients with BED.

#### 2.5.4. Obesity

Obesity is one of the most common health problems and has been extensively studied in neuroscience. In a meta-analysis of 22 neuroimaging studies, Devoto et al. found that patients with obesity showed increased activity in brain regions involved in processing taste and the reward value of food-related stimuli, such as the ventral striatum and insula [106]. These results suggest that obesity might be better explained by the hypersensitivity of the brain involved in reward processing than deficits in reward processing of food in the brain. Recent evidence also shows that even in the resting-state, without food cues, liking food is correlated with the functional connectivity of the orbitofrontal cortex and ventromedial PFC, suggesting that the evaluation of reward and pleasantness of food in the brain is key to understanding and treating patients with obesity [107]. In addition to functional activity, cerebral blood flow increased in the insula of obese and post-obese patients while tasting a liquid meal compared to lean participants, suggesting that abnormal neural responses to food might persist after weight loss in patients with obesity [108]. Moreover, patients with obesity also showed significantly less activity in the dorsolateral PFC after the consumption of a liquid meal [109] and reduced gray matter density in prefrontal areas [110], compared to lean participants, suggesting inhibitory control might be impaired in patients with obesity. Moreover, healthy food choices [44] and successful weight loss in patients with obesity are significantly related to the functional connectivity between the dorsolateral and ventromedial PFC [111]. Based on these results, real-time neurofeedback using fMRI-based functional connectivity between the dorsolateral and ventromedial PFC was applied to patients with obesity, and the results showed that neurofeedback training significantly reduced high-calorie food choices but did not reduce snack intake [112].

In conclusion, abnormal food intake behavior is complicated and not just influenced by neural properties in patients with obesity. Further research is required on the function of gut hormones and the brain–gut axis in the obese population, in addition to reward and inhibitory processing in the brain. We could pinpoint a target brain area for neuromodulation and neurostimulation therapies if we could determine the various elements and neural activities influencing food intake.

#### 2.5.5. Disorders of Gut–Brain Interaction

Although food ingestion is the most important factor that exacerbates the symptoms and overlap between DGBI and EDs [113], only a few neuroimaging studies have focused on abnormal eating-related characteristics and neural responses to food in patients with DGBI, such as irritable bowel syndrome and FD. Rectal or stomach distention is the most frequently studied neuroimaging test, and visceral hypersensitivity and brain regions responsible for increased sensitivity to mechanical and chemical gut stimuli have been demonstrated in patients with DGBI [114,115]. Using resting-state fMRI before and after the consumption of real food, Lee et al. found an expectancy effect of fat concentration in food on FD symptoms, distinct functional connectivity patterns of the insula after consuming a high- or low-fat meal, and the effect of psychological state on brain activity in the frontal gyrus of patients with FD [42]. However, no effect of fat concentration (high-fat vs. low-fat) on symptom severity was found, which suggests that some food-induced symptoms could be evoked by the cognitive perception of food rather than its actual nutritional composition. In a recent meta-analysis, Mao et al. showed that patients with FD also had increased resting-state activity in the visceral sensation and emotion-processing regions, such as the insula, ACC, thalamus, and motor-related brain regions [116], suggesting that more evidence is required to investigate the characteristics of food-related neural responses in DGBI and the distinct and overlapping neural signatures associated with DGBI compared to EDs and healthy populations.

## 3. Future Implications

In addition to the visual and gustatory aspects of food stimuli, olfactory and textural information also affect eating behavior. Olfactory information, as well as visual information, is crucial for the identification of food, and the texture of food affects its sensory satisfaction [117]. The visual, gustatory, olfactory, and somatosensory information of foods is integrated into the striatum, hypothalamus, and OFC and influences the liking and wanting system, food intake behavior, and emotional and autonomic responses to foods (see [118] for a detailed review). In terms of a multisensory approach, virtual reality could be used as an effective way to provide a naturalistic and rich environment for food cues and training simulation programs. Neuroimaging studies may have significant limitations in accurately reflecting the reality of food cues owing to environmental constraints [5,119]. The establishment of more reliable experimental paradigms that better reflect the reality of food cue exposure and choice is another area for future research. For example, one might anticipate that a participant would act differently in the noisy and constrained MRI machine of a hospital than if they were eating real food. Ledoux et al. suggested that environmental cues trigger food cravings; thus, they can be studied using virtual reality. Indeed, Ledoux et al. used a food cue exposure task with neutral images, virtual reality images, and real food cues and found that the virtual reality food cues produced greater food cravings than the neutral cues but lower cravings than the real food cues [120]. Although the results do not demonstrate a superior effect of using virtual reality food cues over real food in terms of subjective craving for food, the use of virtual reality in neuroimaging studies involving food is still desirable. For example, more multisensory cues (olfactory, somatosensory, and even gustatory) could be suggested to participants as virtual reality technology advances. Moreover, virtual reality could be used as part of an inhibitory control training program. Manasse et al. tested the feasibility of using a virtual reality program for inhibitory control training in participants who had recently experienced loss-of-control eating episodes and found that it could potentially reduce loss-of-control eating behavior [121]. Although the above two studies did not show a superior effect of using virtual reality in studying food-related cognitive factors and improving eating behavior, further studies are needed to develop new treatment methods using advanced techniques (e.g., software as a medical device) for patients with an ED.

In addition to virtual reality, advanced neuroimaging techniques may enable more natural feeding settings in the future. We believe that fNIRS will allow for more flexible and realistic experiments in patients with eating disorders because it does not require participants to be supine, nor does it restrict their head motion or metal objects in the body. Both fNIRS and MEG allow participants to sit rather than lie down, and solid foods can be delivered to participants more safely than MRI and PET scanners, which limit participants’ positions to supine. Furthermore, because wearable and wireless fNIRS allow participants to move freely [122,123], it may be possible to evaluate neural activities during natural eating while taking the effects of swallowing and chewing into account [124,125].

It is well known that patients with an ED show abnormal reward responsiveness and disrupted inhibitory control. For example, obese individuals have less effective food-specific inhibitory control and impaired activity/connectivity of inhibitory brain areas (e.g., dorsolateral PFC, inferior, superior, and medial frontal gyri) [56,126]. Thus, although more research is needed to support its effectiveness, new treatment methods targeting inhibitory control, such as behavioral training [127,128], TMS [129,130], tDCS [105,131,132], and neurofeedback techniques [111,133] have emerged as effective approaches. In particular, the dorsolateral PFC has been most frequently used for TMS and tDCS treatments and has also been used as a target region for neuromodulation and neurofeedback treatments [134], suggesting a crucial role of the dorsolateral PFC in cognitive control and emotional regulation, which are related to food cravings and altered eating behaviors [56]. As studies increasingly suggest the importance of new treatment strategies based on neuroimaging findings of potential brain biomarkers, combining neurostimulation and neuromodulation techniques with neuroimaging approaches might allow us to successfully develop effective treatments and demonstrate their neural mechanisms in patients with an ED.

Lastly, in addition to approaches that target the modulation of neural activity and connectivity, changing one’s mindset may have a powerful effect on eating behavior. As mentioned above, eating behavior is not solely determined by energy state and neural properties; our mind can have a significant impact on it. The effects and mechanisms underlying mindset in eating behavior have only recently been studied. Hege et al. found that healthy and lean participants reduced their food size when considering eating for health, which was related to increased activity of the PFC [135], and a similar effect was found in patients with obesity [136]. In future studies, various strategies to create a healthier food-related mindset (e.g., changing food-related environments, food-related instructions, and physical activity) should be developed, and the effects should be tested in patients with an ED.

## 4. Conclusions

Neuroimaging has great potential to provide insight into the neural response to food stimuli. Remarkable advances have been made in understanding the neural activity underlying food perception, not only in normal eating but also in obesity and EDs in recent decades. Previous studies on obesity, EDs, and DGBI have suggested the presence of abnormal brain function in affected people compared to healthy controls (e.g., abnormal impulsivity related to diminished PFC activity, reward and hormonal changes in the gut signaling to the brain via the gut–brain axis). New therapies (e.g., neurofeedback and neurostimulation techniques) have been developed that target the malfunctioning brain regions in patients with an ED, especially reward processing regions (to treat the sensitivity of the reward circuit) and prefrontal areas (to support cognitive control). Future research on functional neuroimaging should be connected to cutting-edge methodologies like virtual reality, neurostimulation, and neuromodulation, as well as new neuroimaging tools that enable us to examine more natural and realistic ingestive behavior and its interaction with the brain. 

## Data Availability

Not applicable.
